# Shear bond strength of one-step self-etch adhesives to dentin: Evaluation of NaOCl pretreatment

**DOI:** 10.4317/jced.54552

**Published:** 2018-02-01

**Authors:** Marco Colombo, Riccardo Beltrami, Marco Chiesa, Claudio Poggio, Andrea Scribante

**Affiliations:** 1 Department of Clinical-Surgical, Diagnostic and Pediatric Sciences – Section of Dentistry, Policlinico “San Matteo”, Piazzale Golgi, Pavia, Italy

## Abstract

**Background:**

The aim of this study was to evaluate the influence of dentin pretreatment with NaOCl on shear bond strength of four one-step self-etch adhesives with different pH values.

**Material and Methods:**

Bovine permanent incisors were used. Four one-step self-etch adhesives were tested: Adper™ Easy Bond, Futurabond NR, G-aenial Bond, Clearfil S3 Bond. One two-step self-etch adhesive (Clearfil SE Bond) was used as control. Group 1- no pretreatment; group 2- pretratment with 5,25 % NaOCl; group 3- pretreatment with 37 % H3PO4 etching and 5,25 % NaOCl. A hybrid composite resin was inserted into the dentin surface. The specimens were tested in a universal testing machine. The examiners evaluated the fractured surfaces in optical microscope to determine failure modes, quantified with adhesive remnant index (ARI).

**Results:**

Dentin pretreatment variably influenced bond strength values of the different adhesive systems. When no dentin pretreatment was applied, no significant differences were found (*P* >.05) among four adhesives tested. No significant differences were recorded when comparing NaOCl pretreatment with H3PO4 + NaOCl pretreatment for all adhesive tested (*P* >.05) except Clearfil S3 Bond that showed higher shear bond strength values when H3PO4 was applied. Frequencies of ARI scores were calculated.

**Conclusions:**

The influence of dentin pretreatment with NaOCl depends on the composition of each adhesive system used. There was no difference in bond strength values among self-etch adhesives with different pH values.

** Key words:**Dentin, pretreatment, self-etch adhesives.

## Introduction

Modern adhesive systems are classified into etch-and-rinse and self-etch adhesives (1). The techniques differ for the acid-etch step. Etch and rinse adhesive systems require acid-etching to promote dentin and enamel demineralization before monomer infiltration, while self-etch adhesives cause a simultaneous substrate demineralization and monomer infiltration (2,3). The amount of substrate demineralization can be related to the initial pH-value of the adhesive system thus subdividing them into: mild (pH of 2 or more), moderate (pH between 1 and 2) and strong (pH of 1 or below) (1,4,5). The monomer infiltration requires a separate bonding step for two-step adhesive systems or it is combined in a single application for one-step adhesive systems (4).

In literature, various authors discussed the bond strength of self-etch adhesive systems to enamel, showing conflicting results; some studies reported comparable data to that observed with etch-and-rinse systems (6-9), whereas other studies considered them less reliable when bonding to dentin (10-12). Self-etching adhesives were introduced in order to simplify the bonding steps, thus reducing the actual bonding time. Since etching and priming of the dentin surface is done in the same step, the resin monomers penetrate the whole depth of the demineralized dentin. Incomplete resin penetration to this depth will leave an exposed demineralized dentin zone at the base of the hybrid layer. In order to increase the bond longevity for the adhesive restorations, it is very important to eliminate this demineralized dentin zone from the bond structures (13).

Pretreatment with NaOCl has nonspecific proteolytic and disinfectant properties; because of these properties, it is widely used in a variety of dental procedures, such as the treatment of root canals, caries removal, and dentin bonding techniques (14). Many studies have evaluated the effect of sodium hypochlorite treatment on the performance of different adhesive systems to dentin (15-18). It was found that sodium hypochlorite application either had no effect, or affected the performance of the different dentin adhesives (improving or reducing bonding performance) (19-21). The use of sodium hypochlorite after etching of the dentin surface was also found to remove the exposed collagen fibers that altered the dentin surface characterization (22). In fact, NaOCl applied to smear layer-covered dentin promotes the dissolution of the organic phase and the smear layer is significantly thinned (14,23). However remnants of super-oxide radicals generated by NaOCl within the dentin substrate inhibit polymerization of resin monomers thus reducing the bond-strength of adhesive systems (16).

The aim of this study was to evaluate the influence of dentin pretreatment with NaOCl on shear bond strength of four one-step self-etch adhesives with different pH values.

## Material and Methods

-Specimens’ preparation

One hundred and fifty bovine permanent incisors freshly extracted and stored in a solution of 0.1% (wt/vol) thymol were used as a substitute for human teeth (24,25). A criterion for tooth selection included intact buccal enamel with no cracks caused by extraction. The teeth were cleansed of soft tissue and embedded in self-curing, fast-setting acrylic resin (Rapid Repair, DeguDent GmbH, Hanau, Germany). Specially fabricated cuboidal Teflon mould were filled with the acrylic resin and allowed to cure, thus encasing each specimen while allowing the buccal surface of dentin to be exposed. Each tooth was oriented so that its labial surface was parallel to the shearing force. The teeth were sectioned parallel to the occlusal surface to expose midcoronal dentin. The exposed dentin surfaces were wet abraded using an automated polishing machine (APL-4; Arotec S.A. Ind Com, Cotia, SP, Brazil) with a 600-grit silicon carbide abrasive paper (SiC) disks for 5 seconds, to obtain a flat and uniform dentin surface. The teeth were randomly assigned into three groups (each made of 50 specimens) according to different dentin surface pretreatments.

Group 1 - Control: no pretreatment was applied; the adhesives were applied according to the manufactures directions.

Group 2 - Pretreatment with 5,25 % NaOCl (Niclor 5; Ogna Laboratori Farmaceutici, Muggiò, Italy) application for 2 min; after rinsing and drying the specimens for 30 seconds each, the adhesives were applied according to the manufacturer’s directions.

Group 3 - Pretreatment with 37% H3PO4 etching (Total Etch; Ivoclar Vivadent AG, Schaan, Liechtenstein) for 15 seconds and 5,25 % NaOCl (Niclor 5; Ogna Laboratori Farmaceutici, Muggiò, Italy) application for 2 min; after rinsing and drying the specimens for 30 seconds each, the adhesives were applied according to the manufacturer’s directions. Each group was then divided into five subgroups of ten teeth each according the bonding agent used.

-Materials tested

The materials used in this study included four one-step self-etch adhesives with different pH values: Adper™ Easy Bond (pH=0,9), Futurabond NR (pH=1,4), G-aenial Bond (pH=1,5), Clearfil S3 Bond (pH=2,7). One two-step self-etch adhesive (Clearfil SE Bond/pH=2,1) was used as control. The specifications of all adhesive systems are listed in  Table 1.

**Table 1:**
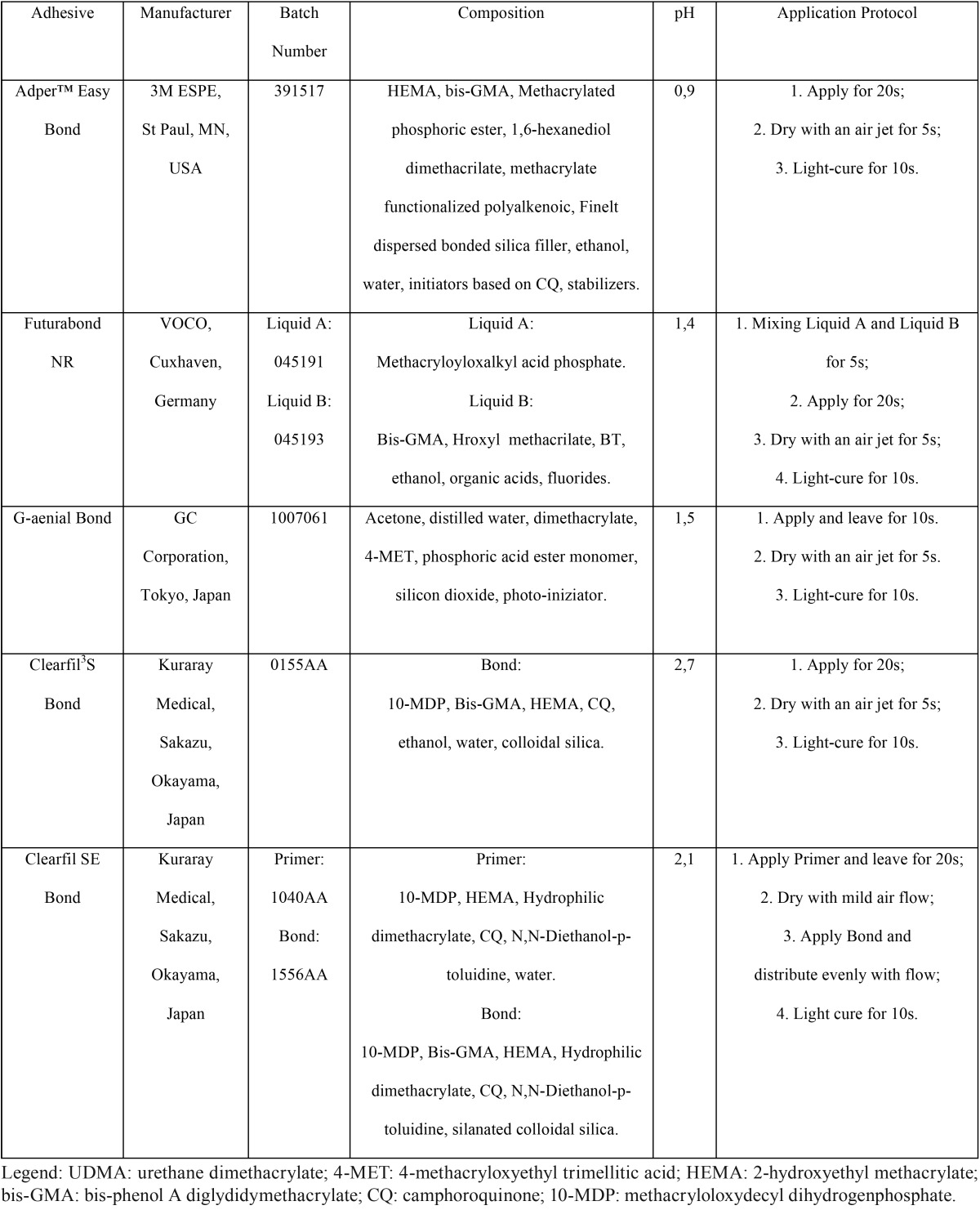
Adhesive systems tested.

-Application of adhesive systems

The adhesive systems were applied to the demarcated bonding area. All adhesives were cured using a LED curing light in soft start-polymerization mode (Celalux 2 High-Power LED curing-light, Voco GmbH, Cuxhaven, Germany) for the times suggested by the manufacturers at a irradiance of 1000 mW/cm2. After adhesive systems application, a hybrid composite resin (Grandio, Voco GmbH, Cuxhaven, Germany) was carefully inserted into the dentin surface by packing the material into cylindrical-shaped plastic matrices with an internal diameter of 2 mm and a height of 2 mm. Excess composite was carefully removed from the periphery of the matrix with an explorer. The composite was cured with an LED curing light in soft start-polymerization mode (Celalux 2 High-Power LED curing-light, Voco GmbH, Cuxhaven, Germany) for 20 seconds at a light intensity of 1000 mW/cm2: the composite buildups were created. Following polymerization, specimens were stored in distilled water for 24 hours at 37°C.

-Shear bond strength testing

After storing, the specimens were tested in a universal testing machine (Model 3343, Instron Corporation, Norwood, MA, USA). Specimens were secured in the lower jaw of the machine so that the bonded cylinder base was parallel to the shear force direction. The tensile bond strength was performed at 0.5 mm/minute until the sample rupture. Specimens were stressed in an occluso-gingival direction at a crosshead speed of 1 mm/min (26-28). The maximum load necessary to debond was recorded in Newton (N) and calculated in MPa as a ratio of Newton to surface area of the cylinder. After the testing procedure, the fractured surfaces were examined in optical microscope (Stereomicroscope SR, Zeiss, Oberkochen, Germany) at a magnification of 10X to determine failure modes and classified as adhesive failures, cohesive failures within the composite, or cohesive failures within the tooth (29). The adhesive remnant index (ARI) was used to assess the amount of adhesive left on the dentin surface (30). This scale ranges from 0 to 3. A score of 0 indicates no adhesive remaining on the tooth in the bonding area; 1 indicates less than half of the adhesive remaining on the tooth; 2 indicates more than half of the adhesive remaining on the tooth; and 3 indicates all adhesive remaining on the tooth. The ARI scores were used as a method of defining bond failure site among the dentin, the adhesive, and the composite.

-Statistical analysis

Statistical analysis was performed with Stata 9.0 software (Stata, College Station, Tx, USA). Descriptive statistics, including the mean, standard deviation, median, and minimum and maximum values were calculated for all groups. Kolmogorov and Smirnov (KS) test was applied to assess normality of distributions. An analysis of variance (two-ways ANOVA) was applied to determine whether significant differences in debond values existed among the groups. The Dunn test was used as post-hoc. The chi-squared test was used to determine significant differences in the ARI scores among the different groups. Significance for all statistical tests was predetermined at *P*<.05.

## Results

**Figure 1:**
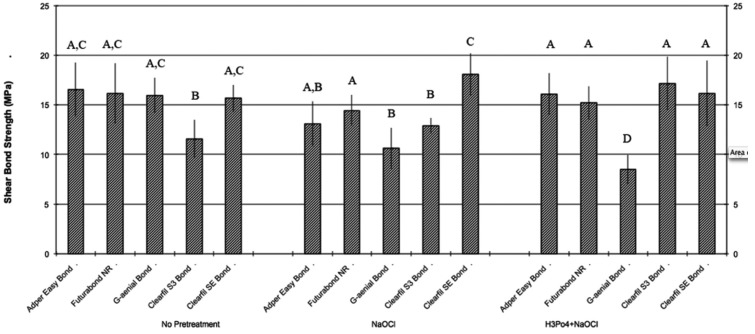
Mean shear bond strength and standard deviations of the different groups.

**Figure 2:**
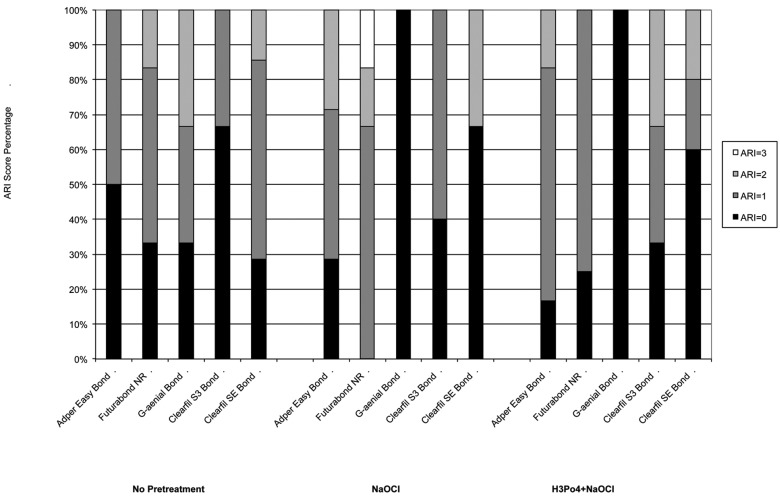
Distribution of ARI scores of the different groups.

Descriptive statistics of the shear bond strength (MPa) of the different groups are illustrated in  Table 2 and in Fig. 1. KS test assessed normal data distributions (*P*>0.05). ANOVA showed the presence of significant differences among the various groups (*P*<.001) as reported in  Table 3. Post hoc Dunn test showed that when no dentin pretreatment was applied, no significant differences were found (*P*>.05) among four different adhesives tested (AdperTM Easy bond, Futurabond NR, G-aenial Bond and Clearfil SE Bond) and that all showed significantly higher shear bond strength values than Clearfil S3 Bond (*P*<.01). Moreover, when NaOCl pretreatment was conducted four adhesives tested (AdperTM Easy bond, Futurabond NR, Clearfil S3 Bond and Clearfil SE Bond) showed no significant differences in shear bond strength values when compared with untreated groups (*P*>.05), whereas when testing G-aenial Bond strength values were significantly lower than those recorded under untreated dentin (*P*<.001). Correspondly, when H3PO4 + NaOCl pretreatment was tested three adhesives (AdperTM Easy bond, Futurabond NR and Clearfil SE Bond) showed no significant differences in shear bond strength values when compared with untreated groups (*P*>.05), whereas when testing G-aenial Bond strength values were significantly lower than those recorded under untreated dentin (*P*<.001) while Clearfil S3 Bond showed significantly higher values (*P*>.05). Finally, no significant differences were recorded when comparing NaOCl pretreatment with H3PO4 + NaOCl pretreatment for all adhesive tested (*P*>.05) except Clearfil S3 Bond. When comparing ARI Score results of the different groups no statistical difference was found in frequency distribution among various groups, that all showed a significant prevalence of ARI Score of “0” and “1”, as illustrated in Fig. 2.

**Table 2:**
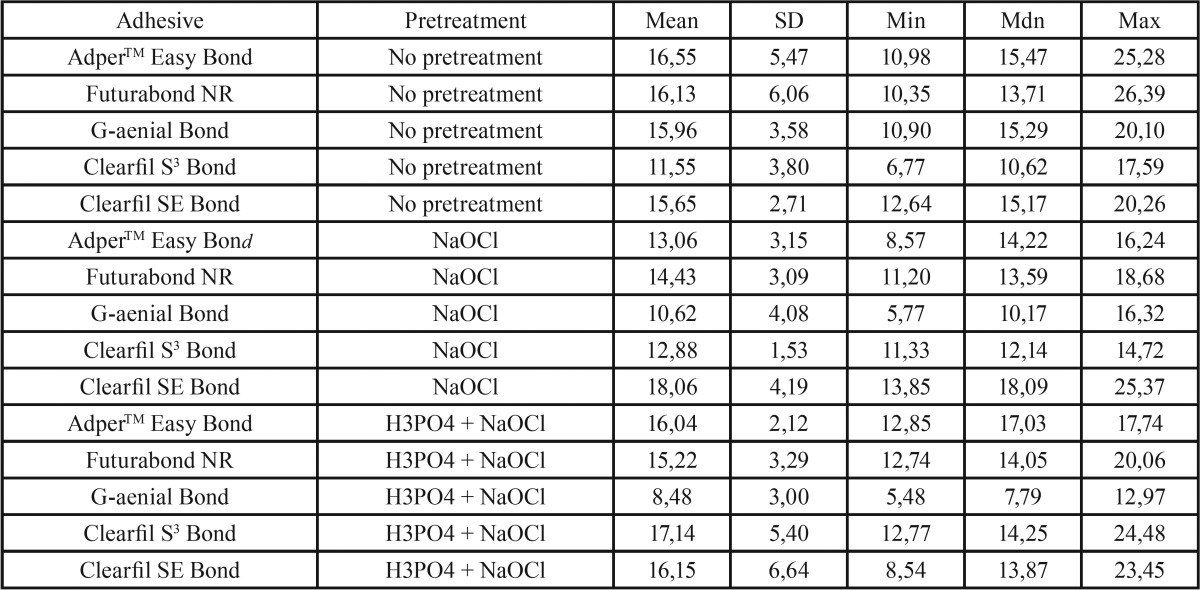
Descriptive statistics (in MPa) of shear bond strengths of the 10 subgroups tested (each subgroup consisted of 10 specimens). SD: Standard deviation.

**Table 3:**
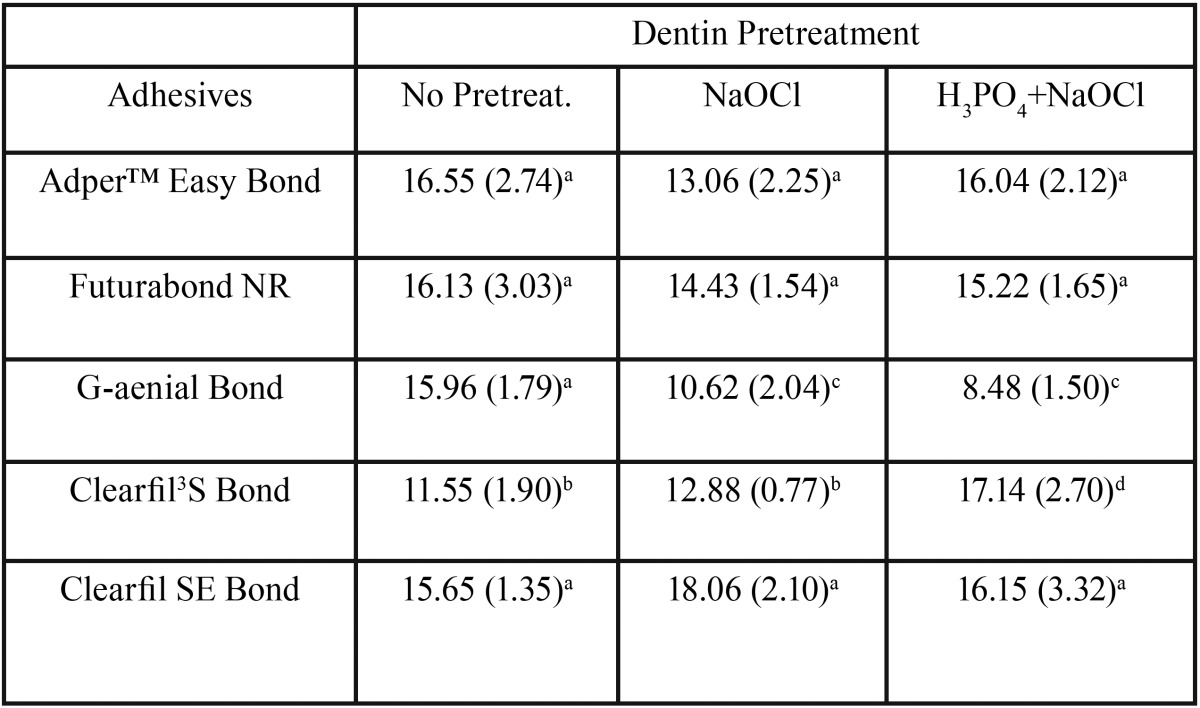
Mean and standard deviation (in parenthesis) in MPa for each material tested. The same superscript letter in vertical row indicate no significant differences (*P*&0.05).

## Discussion

In order to compare data from the current study with that reported in previous bovine dentin bond strength tests, bovine teeth were used as a substitute for human teeth in the current study. Bovine teeth have large, flat surfaces and are unlikely to have undergone prior caries challenges that could affect the test result. The mineral distribution within the carious lesions in bovine teeth is reported to be similar to human teeth, and the structural changes that occur in human and bovine teeth are also similar (31,32). Modern one-step self-etch adhesives simplify the technique and reduce the number of clinical steps but substrate pretreatment could influence their bond strength values (33-35). The effect of such additional pretreatment on dentin bond strength is also controversially discussed in the literature. Its use might be beneficial with some self-etching adhesives, but this depends largely on the properties of the adhesive itself.

Differently from other studies, the present research focused on normal dentin because in the clinical practice there is no need to leave caries-affected dentin. Moreover it has been demonstrated a significantly lower shear bond strength in caries-affected dentin than in normal dentin using one-step and two-step self-etching adhesives (36); in fact the acid-resistant minerals within the caries-affected dentin smear layer, which are derived from the occluding mineral deposits within the dentinal tubules, might interfere with dissolution of the smear layer by the self-etch adhesives (37).

As reported in Fig. 1, AdperTM Easy Bond, Futurabond NR, Clearfil S3 Bond and the control Clearfil SE Bond maintained shear bond strength constant both after pretreatment with 5,25 % NaOCl application for 2 min, both after pretreatment with 37% H3PO4 etching for 15 seconds and 5,25 % NaOCl for 2 min. Differently, G-aenial Bond showed statistical significant reduction of shear bond strength after pretreatment. These results are in contrast with the current concepts on resin/dentin adhesion which suggest that bonding to dentin prior to use of self-etching primers would be more predictably achieved by first removing the smear layer with a separate proteolytic conditioning step (38). The lower shear bond strength obtained by G-aenial Bond evaluated after NaOCl treatment may have been caused by the association of presence of an additional demineralization induced by the self-etching functional monomers, occurrence of residual glycosaminoglycans components of the organic matrix (which are resistant to strong acids and NaOCl) and disruption by NaOCl of pyridinoline cross-links that occur in the Type I dentin collagen, with the formation of chloramines and protein derived radical intermediates (39).

Reactive radicals could interfere with vinyl free-radicals emitted during photo-polymerization thus reducing the conversion from monomer to polymer (39). For the remnant adhesives tested the application of pretreatments did not influence the shear bond strength even if it contributed with non-specific proteolytic properties in the reduction of the smear layer due to the dissolution of the organic phase. Clearfil S3 Bond showed a significant increase in shear bond strength as reported in Table 3. As manufacturers suggested, this adhesive system presents a Molecular Dispersion Technology, which enables the two-phase liquids of hydrophilic and hydrophobic components to be maintained in a homogeneous state even when the solvent is evaporated, improving bond quality. This aspect is well confirmed by the results of the ARI scores; as showed in Fig. 2, the failure of the adhesion when pretreatment is applied to Clearfil S3 Bond is significantly higher among the adhesive. When shear bond strength is reported to be significantly lower, as for G-aenial Bond, the ARI score when pretreatment is applied indicates in all cases not a cohesive failure, but a failure of the adhesion at the interface between dentin and the adhesive system (40).

## Conclusions

The pretreatment with H3PO4 and NaOCl enhanced the shear bond strength of AdperTM Easy Bond, Futurabond NR, Clearfil S3 Bond and Clearfil SE Bond; but the differences between the values were not statistically significant. Differently, the pretreatment reduced significantly the shear bond strength of G-aenial Bond. Self-etch adhesives failed primarily in the adhesive substrate (ARI = 0 and ARI=1), without significantly differences among the various groups.
